# Microelastic mapping of the rat dentate gyrus

**DOI:** 10.1098/rsos.150702

**Published:** 2016-04-20

**Authors:** Tomás Luque, Michael S. Kang, David V. Schaffer, Sanjay Kumar

**Affiliations:** 1 Unit of Biophysics and Bioengineering, School of Medicine, University of Barcelona, Barcelona, Spain; 2 Department of Bioengineering, University of California, Berkeley, CA 94720, USA; 3 UC Berkeley – UCSF Graduate Program in Bioengineering, University of California, Berkeley, CA 94720, USA; 4 Department of Chemical and Biomolecular Engineering, University of California, Berkeley, CA 94720, USA; 5 Helen Wills Neuroscience Institute, University of California, Berkeley, CA 94720, USA

**Keywords:** neural stem cells, atomic force microscopy, hippocampus, elastic modulus

## Abstract

The lineage commitment of many cultured stem cells, including adult neural stem cells (NSCs), is strongly sensitive to the stiffness of the underlying extracellular matrix. However, it remains unclear how well the stiffness ranges explored in culture align with the microscale stiffness values stem cells actually encounter within their endogenous tissue niches. To address this question in the context of hippocampal NSCs, we used atomic force microscopy to spatially map the microscale elastic modulus (*E*) of specific anatomical substructures within living slices of rat dentate gyrus in which NSCs reside during lineage commitment *in vivo*. We measured depth-dependent apparent *E*-values at locations across the hilus (H), subgranular zone (SGZ) and granule cell layer (GCL) and found a two- to threefold increase in stiffness at 500 nm indentation from the H (49 ± 7 Pa) and SGZ (58 ± 8 Pa) to the GCL (115 ± 18 Pa), a fold change in stiffness we have previously found functionally relevant in culture. Additionally, *E* exhibits nonlinearity with depth, increasing significantly for indentations larger than 1 µm and most pronounced in the GCL. The methodological advances implemented for these measurements allow the quantification of the elastic properties of hippocampal NSC niche at unprecedented spatial resolution.

## 1. Introduction

Neural stem cells (NSCs), which reside in the hippocampus and the subventricular zone of the adult mammalian brain, continually generate neurons throughout adult life [1,2]. Hippocampal NSCs reside in the subgranular cell layer (SGZ) of the dentate gyrus (DG), undergo self-renewal and neuronal differentiation throughout adult life and play key roles in learning and memory. Differentiation is a coordinated process involving migration over several cell diameters into the granule cell layer (GCL), extension of axons through the hilus (H) and into CA3, and development of dendrites through the GCL and into the molecular layer [1,3,4].

Importantly, this process is not static but dynamically responds to numerous cellular and molecular inputs. For instance, key biochemical signalling factors within the SGZ microenvironment have been found to regulate both neural stem and progenitor cell proliferation—such as sonic hedgehog [5], fibroblast growth factor-2, heparin-binding EGF-like growth factor [6] and vascular endothelial growth factor [7]—and neuronal differentiation—such as Wnt3a [8,9] and ephrin-B2 [10]. Additionally, not only the biochemical but also the biophysical microenvironment may regulate stem cell function. For example, mechanical signalling through cell–extracellular matrix (ECM) interactions has been shown to play a role in regulating NSC differentiation. In particular, the elastic modulus (*E*) of the ECM substrate has been shown to regulate NSC lineage *in vitro* [11,12], with soft ECMs promoting NSC neurogenesis and stiff ECMs suppressing it [12,13]. Despite the clear instructive effects of elastic modulus on stem cell behaviour *in vitro*, and the *in vivo* regulation of stem cell behaviour by signals that are modulated *in vitro* by stiffness differences [13], the degree to which *E* may vary in the *in vivo* niche is unknown.

Atomic force microscopy (AFM) indentation has facilitated direct measurement of *E* with higher (up to sub-micrometre) spatial resolution compared with other methods such as optical coherence tomography [14] and stress-relaxation micro-indentation [15], and has been used notably by Morrison and co-workers to map and report stiffness across the rat hippocampus bulk from the CA1 to CA3 pyramidal cell layers and across the DG [16]. Such measurements have informed the design of *in vitro* assay platforms capable of recapitulating *in vivo* behaviour. Synthetic polyacrylamide matrix culture systems have shown stiffness-dependent instruction of NSC differentiation [12,13], neurogenic instruction of pluripotent stem cell differentiation [17] and full neuronal maturation and subtype differentiation with physiological stiffness values [18]. That said, these seminal AFM indentation measurements focused on large size scale variations in stiffness across the entire hippocampus, rather than high-resolution investigation of the specific regions in which hippocampal NSCs reside. While technically challenging, obtaining higher resolution mechanical maps of portions the hippocampal NSC niche relevant to neurogenesis could provide valuable insight into potential mechanical influences on NSCs during proliferation and differentiation *in vivo*. To address this unmet need, we conducted AFM measurements of the elastic modulus of the DG between the GCL and H, a region that directly encompasses the stem cell niche.

## 2. Results

### 2.1. Preparation of hippocampal slices for atomic force microscopy stiffness mapping

**Figure 1.**
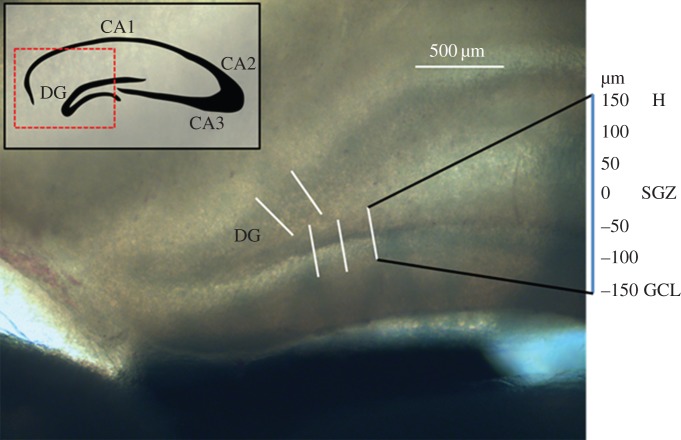
Transmitted light image of a representative coronal hippocampal section. Measurements were taken in the DG, at 7 points along a 300 µm linear profile from the GCL to the H. Five profiles for each slice were taken, and representative measurement locations are indicated.

To maximally preserve physiological biomechanical properties of brain tissue, we designed a protocol for much more rapid (less than 1 h after sacrifice), fixative-free AFM measurements of hippocampal slices. Briefly, we extracted brains from freshly sacrificed rats and immersed them in 0°C cutting solution. We used a vibratome to generate 400 µm thick coronal sections, and quickly transported sections to the AFM stage. To save time that would normally be needed to allow adhesive bonding of the sample to the substrate, we secured the slices by placing them under a nylon mesh secured by a metal ring. A compliant nylon mesh with wide spacing (2 mm) was used to minimize tissue deformation and strain near the point of measurement. AFM measurements were performed between 30 and 60 min after sacrifice (figure 1). Importantly, our protocol uses no chemical fixatives and minimal embedding, and the AFM tip is allowed to directly contact the tissue surface. A thick sample (400 µm) and small indentation depths (less than 2 µm) were used to prevent the underlying polystyrene dish from contributing to measurements.

### 2.2. Stiffness is variable across the stem cell niche

**Figure 2.**
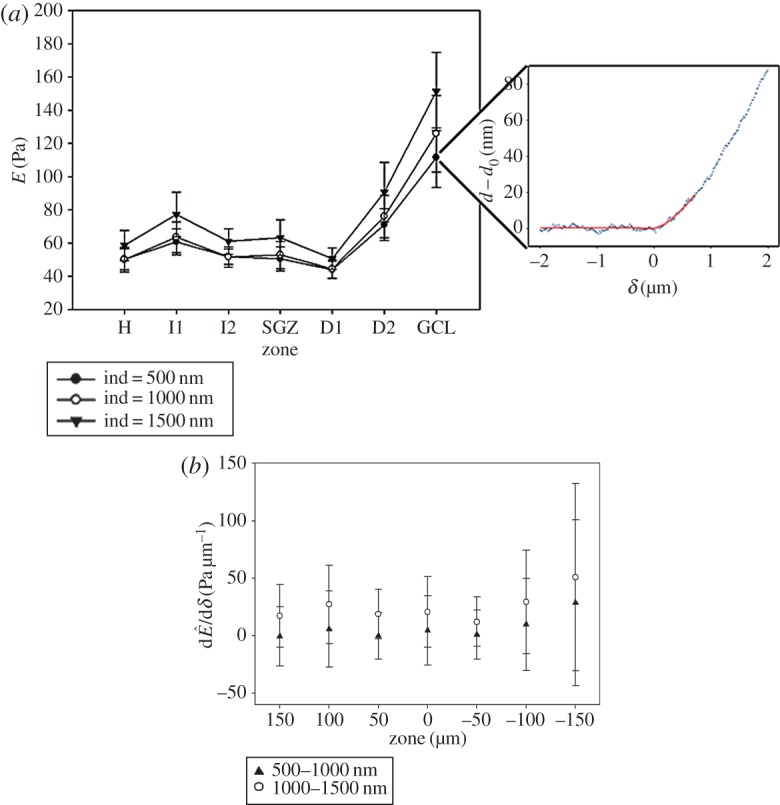
(*a*) Average *E* from six brain samples with representative force curve. In the inset representative force curve, points depict experimental data for *z*, cantilever vertical position and *d*, cantilever deflection, and the trend line is a two-part fitted model: a straight line for the non-contact zone and a 3/2 power of *δ* for the contact region. In the graph, each point is the average of five force curves, and shown for three different indentation depths. The tissue is slightly nonlinear with indentation depth. Standard error is indicated. Stiffnesses in H and SGZ are significantly different from GCL (*p* < 0.05). SGZ and GCL both showed significant differences between *δ* = 1000 nm and *δ* = 1500 nm. SGZ showed significant difference between *δ* = 500 nm and *δ* = 1000 nm. (*b*) Graph of derivative of *Ê* with respect to indentation depth, indicating the depth dependence of strain stiffening.

Five linear profiles of force curves were acquired in six samples (figure 1), up to an estimated indentation of 2 µm. The contact point was algorithmically estimated, and force curves were fitted to the Hertz contact model for a sphere indenting up to a maximum indentation (*δ*_max_) of 500 nm. Owing to the large polystyrene bead (25 µm) used as a tip, measurements incorporate both cell and matrix mechanical properties. Two iterations of a nonlinear fit were used to obtain *E* (figure 2*a*). The mean at 500 nm indentation was 49 ± 7 Pa (mean ± s.e., *N* = 6) for the H, 58 ± 8 Pa in the SGZ and 115 ± 18 Pa in the GCL (figure 2*a*). These values are comparable with previous reports of *E* as approximately 100 Pa in the DG for a 500 nm indentation [16]. However, we discovered that the GCL stiffness was significantly (*p *< 0.01, two- to threefold increase) higher than the SGZ and the H, whereas comparisons between the SGZ and H showed no significant difference (*p* > 0.05). The measurements thus indicate a gradient of increasing stiffness between the SGZ and the GCL (figure 2*a*), which has not been previously observed.

### 2.3. Dentate gyrus stiffness exhibits slight nonlinearity for indentations greater than 1 µm

Tissue behaviour (linear or nonlinear) at supraphysiological strains is a critical component for understanding response to injury, and the hippocampus in particular is uniquely sensitive to mechanical strain [19]. We therefore computed a pointwise, depth-dependent apparent Young's modulus (*Ê*) at indentations of 0.5, 1 and 1.5 µm. At a greater indentation depth, the sample exhibited higher stiffness values than at lower depths for particular locations. Specifically, *Ê* (*δ* = 500 nm) was 50 ± 6 Pa (H), 51 ± 7 Pa (SGZ) and 111 ± 18 Pa (GCL), but with *δ* = 1500 nm these values increased to 59 ± 9 Pa, 63 ± 11 Pa and 151 ± 24 Pa, respectively (figure 2*a,b*). Both the SGZ and the GCL exhibited significant differences (*p* < 0.05) in *Ê* between 1 and 1.5 µm indentations, but only the SGZ showed a difference between 0.5 and 1 µm. While tissue nonlinearity has been previously reported for the DG bulk as between 90 and 130 Pa [16], differences in nonlinearity have not been observed within the sub-anatomical regions of the hippocampus and NSC niche.

## 3. Discussion

In this study, we spatially mapped the elastic modulus of the hippocampal NSC niche using AFM. Our work both represents a key methodological advance and reveals new insight into the micromechanical properties of the endogenous NSC niche, thereby providing crucial design input for systems to recapitulate NSC neurogenesis *in vitro*. Methodologically, our approach is expected to preserve key mechanical properties of brain tissue by employing no chemical fixation and only minimal embedding, as well as by reducing preparation time by physically anchoring rather than adhesively bonding the tissue to the substrate. Additionally, our approach incorporates automated identification AFM tip contact points, similar to methods previously reported [20,21], which have typically been identified by visual examination. This invites error and observer bias that may significantly affect the resulting curve fit and extraction of *E* and *Ê*. While important advances have been made to automate this process through the use of algorithmic models, these approaches rely on empirically determined criteria to define the contact point. Moreover, these models are suited for samples at least an order of magnitude stiffer than brain tissue and have difficulty identifying contact points for very soft gels <3 kPa [22]. To remedy this concern, we used an algorithmic, iterative fitting procedure that defines a contact point based on contact theory, which allowed us to identify the contact point more reproducibly and systematically.

These advances in sample preparation, together with the high spatial resolution of AFM, allowed us to spatially map DG substructures and identify differences in stiffness across regions of the DG, which has not been yet evaluated with high resolution. The SGZ, where the progenitor pool is maintained, and the H, through which new axons grow and project to the CA3, both show stiffness near 50 Pa. The GCL, through which newly differentiated neurons migrate and extend dendrites, shows twofold higher stiffness. The difference in stiffness in the GCL may be due to the densely packed mature granule cells, whereas the lower stiffness in the SGZ and H may be due to differences in cell body density, or even possibly different mechanical properties of immature progenitors. These measurements show that among these anatomical features, there are stiffness gradients that are in a position to influence cell differentiation and axonal and dendritic growth. Additionally, we have identified substructure specific nonlinear stiffening for indentations higher than a micrometre, which indicates possible heterogeneities between tissue substructures, such as differences in cell body and nuclei density between regions. Previous work has identified the hippocampus bulk as possessing strain-stiffening properties, we have shown and characterized precise values for the DG, and notably see this behaviour in the SGZ and GCL but not in the H. While these results are compatible with the observation that many biopolymers can exhibit strain-stiffening behaviour, the reason for this and its physiological implications are unknown and may be the subject of future work [23]. Finally, synthetic systems used to study NSC differentiation have to date been uniform in stiffness, and future work with new culture platforms may explore whether microscale stiffness gradients could serve an informative role in neural stem cell development.

Our measurements also validate the physiological relevance of engineered ECM substrates to drive NSC neurogenesis in culture. We show that the stiffness of living DG tissue lies within a range of 50–150 Pa, which is consistent with past AFM measurements of bulk hippocampal tissue [16] and closely matches stiffness regimes previously identified (200 Pa in two-dimensional culture [12,13] and 180 Pa in three-dimensional culture [24]) to be pro-neurogenic *in vitro* for NSCs, mesenchymal stem cells [11] and human pluripotent stem cells [17]. However, it is important to note some caveats that accompany our measurements. While reducing sample preparation time and removing fixation steps are expected to preserve physiological properties of brain, indentation measurements are still taken on a cut surface of a brain slice. This can cause significant cell death and relaxation in the brain matrix in the plane of indentation. Additionally, in order to better preserve tissue properties, measurements were obtained in buffer at refrigerant temperature (4°C) rather than body temperature, which may produce some artefactual stiffening due to tissue contraction. Last, our study examines only juvenile rats at P18–22, while neurogenesis is an ongoing process, especially in adults. Indeed, the stiffness of bulk hippocampus has been shown to vary by more than 100% between P10 and adulthood [25]. Nevertheless, our measurements therefore offer key, novel validation to these materials strategies by demonstrating that these pro-neurogenic stiffness values match well with the stiffness of microscale regions associated with NSC proliferation (SGZ) and differentiation (GCL) in the hippocampus.

It is anticipated that further methodological refinements may enable even higher-resolution mapping of these niches, which in turn may yield more sophisticated input into the design of neurogenic culture scaffolds. Additionally, it would be useful to revisit these measurements with specific hippocampal disease models to investigate potential biomechanical changes in the NSC niche that may influence neurogenesis in currently unappreciated ways. Finally, by coupling these AFM measurements with live cell markers that track specific stages of neural lineage commitment maturation, it may be possible to correlate changes in microscale tissue mechanics within the DG with the dynamics of neurogenesis. With the emergence of increasingly sophisticated materials strategies that enable patterning of ECM stiffness in time and space, such measurements help the field discover and recapitulate subtleties of the NSC niche.

## 4. Material and methods

### 4.1. Murine hippocampus preparation

Coronal hippocampal sections were prepared as described previously [26]. Briefly, juvenile (p18–p22) Sprague–Dawley rats were anaesthetized with isoflurane and sacrificed by decapitation. The brain was extracted and kept in a low-sodium, bicarbonate-buffered cutting solution at ambient temperature with constant bubbling with 95% O_2_ and 5% CO_2_, and 400 μm coronal sections were cut with a Leica VT1000S vibratome at 0°C. The sections were stored in Ringer's solution at ambient temperature and bubbling with 95% O_2_ and 5% CO_2_ and were quickly transported to the AFM stage. Sections were gently weighed down for measurement on a Petri dish with parallel nylon fibres (approx. 2 mm spacing) attached to a metal ring, and measured within 30–60 min after sacrifice. Cutting solution composition was 75 mM sucrose, 85 mM NaCl, 25 mM NaHCO_3_, 25 mM d-(+)-glucose, 2.5 mM KCl, 1.25 mM NaH_2_PO_4_–H_2_O, 0.5 mM ascorbic acid, 4 mM MgCl_2_, 0.5 mM CaCl_2_, 320 mOsm l^−1^, pH 7.2.

### 4.2. Atomic force microscopy measurements

Indentation measurements were obtained with a NanoScope Catalyst (Bruker Corporation, Billerica, MA, USA) atomic force microscope mounted on an inverted optical microscope (Eclipse Ti-E, Nikon Corporation, Chiyoda, Tokyo, Japan). A 25 µm diameter polystyrene bead (Polysciences, Inc., Warrington, PA, USA) was epoxied to the end of a soft (*k* = 0.01 N m^−1^) tipless cantilever (MLCT-O10, Bruker). The combination of a soft cantilever and a large bead (compared with the indentation) allowed us to measure the small deflections that such soft samples caused on the cantilever. Fabrication was validated with scanning electron microscopy, and cantilever spring constants (*k*) were measured via thermal oscillation [27] using an Asylum MFP-3D Infinity AFM (Asylum Research, Santa Barbara, CA, USA).

For each sample, five linear profiles transverse to the DG layers were acquired (indicated by lines in figure 1). Seven measurements were taken across each profile, with the centre point directly on the SGZ and a lateral spacing of 50 µm. Each measurement was the average of five force curves, obtained with ramp amplitude of 10 µm and frequency of 0.5 Hz, resulting in tip velocity of 10 µm s^−1^.

### 4.3. Data processing

The force *F* exerted on the cantilever was calculated as a Hookean linear spring:

4.1 $$F=k(d-d_{0})$$

and indentation depth *δ* was defined as

4.2 $$\delta =(z-z_{c})-(d-d_{0})$$

with *d* as cantilever deflection, *d*_0_ as deflection offset, *z* as cantilever vertical position, and *z*_c_ as cantilever vertical position at the contact point. These data were fitted to the Hertz contact model for a sphere indenting an infinite sphere using a nonlinear least-squares method [19]:

4.3 $$F=\frac{4\hat{E}}{3(1-v^{2})}R^{1/2}\delta^{3/2}$$

with *R* defined as bead radius (12.5 µm) and *v* as Poisson's ratio. We assumed a Poisson's ratio of 0.5, which agrees closely with simulation [28] and past measurements [29]. We fitted a two-part equation (a line for the non-contact zone and the Hertz elastic model for the contact zone) to the entire dataset using a nonlinear least-squares method (Matlab, The MathWorks, Natick, MA, USA) to obtain an estimation of the contact point (*z*_c_, *d*_0_). We iterated the fit using a defined maximum indentation (*δ*_max_) to refine our estimation of the contact point and *E*. Additional fittings did not appreciably improve the error. We report a pointwise apparent Young's modulus (*Ê*(*δ*)), reported at three chosen indentations (500 nm, 1 µm and 1.5 µm). Statistical analysis was performed with a two-way repeated measures analysis of variance (Sigma Plot, Systat Software), with factors region and indentation.

## Supplementary Material

Computed Young's Modulus from atomic force microscopy data. Sheets 1–6 contain data from 6 different brains, one per sheet. Within each sheet, data from five slices (a-e) are reported. For each location on the slice (−1 to 7), eight locations were probed, with five force curves each (0–4). Each force curve was fit to a maximum indentation depth of 3000 nm (pre500 – i3000 representing indentation depth). All data is compiled in Sheet 7. Cells highlighted in red indicate data of poor quality that was discarded or not collected. Cells highlighted in blue are comments.

## Data accessibility

Elastic moduli from atomic force microscopy curves are available at doi:10.5061/dryad.tv41g.

## References

[1] Deng W, Aimone JB, Gage FH 2010 New neurons and new memories: how does adult hippocampal neurogenesis affect learning and memory? Nat. Rev. Neurosci. 11, 339–350. (doi:10.1038/nrn2822) 20354534 10.1038/nrn2822 PMC2886712

[2] Gage FH 2000 Mammalian neural stem cells. Science 287, 1433–1438. (doi:10.1126/science.287.5457.1433) 10688783 10.1126/science.287.5457.1433

[3] Kempermann G, Gage FH 2002 Genetic influence on phenotypic differentiation in adult hippocampal neurogenesis. Dev. Brain Res. 134, 1–12. (doi:10.1016/S0165-3806(01)00224-3) 11947932 10.1016/s0165-3806(01)00224-3

[4] Santarelli L et al. 2003 Requirement of hippocampal neurogenesis for the behavioral effects of antidepressants. Science 301, 805–809. (doi:10.1126/science.1083328) 12907793 10.1126/science.1083328

[5] Lai K, Kaspar BK, Gage FH, Schaffer DV 2003 Sonic hedgehog regulates adult neural progenitor proliferation in vitro and in vivo. Nat. Neurosci. 6, 21–27. (doi:10.1038/nn983) 12469128 10.1038/nn983

[6] Jin K, Sun Y, Xie L, Batteur S, Mao XO, Smelick C, Logvinova A, Greenberg DA 2003 Neurogenesis and aging: FGF-2 and HB-EGF restore neurogenesis in hippocampus and subventricular zone of aged mice. Aging Cell 2, 175–183. (doi:10.1046/j.1474-9728.2003.00046.x) 12882410 10.1046/j.1474-9728.2003.00046.x

[7] Cao L, Jiao X, Zuzga DS, Liu Y, Fong DM, Young D, During MJ 2004 VEGF links hippocampal activity with neurogenesis, learning and memory. Nat. Genet. 36, 827–835. (doi:10.1038/ng1395) 15258583 10.1038/ng1395

[8] Lie D-C et al. 2005 Wnt signalling regulates adult hippocampal neurogenesis. Nature 437, 1370–1375. (doi:10.1038/nature04108) 16251967 10.1038/nature04108

[9] Kuwabara T et al. 2009 Wnt-mediated activation of NeuroD1 and retro-elements during adult neurogenesis. Nat. Neurosci. 12, 1097–1105. (doi:10.1038/nn.2360) 19701198 10.1038/nn.2360 PMC2764260

[10] Ashton RS, Conway A, Pangarkar C, Bergen J, Lim K, Shah P, Bissell M, Schaffer DV 2012 Astrocytes regulate adult hippocampal neurogenesis through ephrin-B signaling. Nat. Neurosci. 15, 1399–1406. (doi:10.1038/nn.3212) 22983209 10.1038/nn.3212 PMC3458152

[11] Engler AJ, Sen S, Sweeney HL, Discher DE 2006 Matrix elasticity directs stem cell lineage specification. Cell 126, 677–689. (doi:10.1016/j.cell.2006.06.044) 16923388 10.1016/j.cell.2006.06.044

[12] Saha K, Keung AJ, Irwin EF, Li Y, Little L, Schaffer DV, Healy KE 2008 Substrate modulus directs neural stem cell behavior. Biophys. J. 95, 4426–4438. (doi:10.1529/biophysj.108.132217) 18658232 10.1529/biophysj.108.132217 PMC2567955

[13] Keung AJ, de Juan-Pardo EM, Schaffer DV, Kumar S 2011 Rho GTPases mediate the mechanosensitive lineage commitment of neural stem cells. Stem Cells 29, 1886–1897. (doi:10.1002/stem.746) 21956892 10.1002/stem.746 PMC5990277

[14] Lee SJ, King MA, Sun J, Xie HK, Subhash G, Sarntinoranont M 2014 Measurement of viscoelastic properties in multiple anatomical regions of acute rat brain tissue slices. J. Mech. Behav. Biomed. Mater. 29, 213–224. (doi:10.1016/j.jmbbm.2013.08.026) 24099950 10.1016/j.jmbbm.2013.08.026 PMC8011428

[15] Elkin BS, Ilankovan AI, Morrison B 2011 A detailed viscoelastic characterization of the P17 and adult rat brain. J. Neurotrauma 28, 2235–2244. (doi:10.1089/neu.2010.1604) 21341982 10.1089/neu.2010.1604

[16] Elkin BS, Azeloglu EU, Costa KD, Morrison B III 2007 Mechanical heterogeneity of the rat hippocampus measured by atomic force microscope indentation. J. Neurotrauma 24, 812–822. (doi:10.1089/neu.2006.0169) 17518536 10.1089/neu.2006.0169

[17] Keung AJ, Asuri P, Kumar S, Schaffer DV 2012 Soft microenvironments promote the early neurogenic differentiation but not self-renewal of human pluripotent stem cells. Integr. Biol. 4, 1049–1058. (doi:10.1039/c2ib20083j) 10.1039/c2ib20083j PMC3459311 22854634

[18] Keung AJ, Dong M, Schaffer DV, Kumar S 2013 Pan-neuronal maturation but not neuronal subtype differentiation of adult neural stem cells is mechanosensitive. Sci. Rep. 3, 1817 (doi:10.1038/srep01817) 23660869 10.1038/srep01817 PMC3650663

[19] Lin DC, Dimitriadis EK, Horkay F 2007 Robust strategies for automated AFM force curve analysis—I. Non-adhesive indentation of soft, inhomogeneous materials. J. Biomech. Eng. 129, 430–440. (doi:10.1115/1.2720924) 17536911 10.1115/1.2720924

[20] Costa KD 2006 Imaging and probing cell mechanical properties with the atomic force microscope. In Cell imaging techniques: methods and protocols (eds DJ Taatjes, BT Mossman). Methods in Molecular Biology, vol. 319, pp. 331–361. Totowa, NJ: Humana Press. (doi:10.1007/978-1-59259-993-6_17) 10.1007/978-1-59259-993-6_17 16719364

[21] Atkins CM 2011 Decoding hippocampal signaling deficits after traumatic brain injury. Transl. Stroke Res. 2, 546–555. (doi:10.1007/s12975-011-0123-z) 23227133 10.1007/s12975-011-0123-z PMC3514866

[22] Thomas G, Burnham NA, Camesano TA, Wen Q 2013 Measuring the mechanical properties of living cells using atomic force microscopy. J. Vis. Exp. 76, 50497 (doi:10.3791/50497) 10.3791/50497 PMC3729185 23851674

[23] Erk KA, Henderson KJ, Shull KR 2010 Strain stiffening in synthetic and biopolymer networks. Biomacromolecules 11, 1358–1363. (doi:10.1021/bm100136y) 20392048 10.1021/bm100136y

[24] Banerjee A, Arha M, Choudhary S, Ashton RS, Bhatia SR, Schaffer DV, Kane RS 2009 The influence of hydrogel modulus on the proliferation and differentiation of encapsulated neural stem cells. Biomaterials 30, 4695–4699. (doi:10.1016/j.biomaterials.2009.05.050) 19539367 10.1016/j.biomaterials.2009.05.050 PMC2743317

[25] Elkin BS, Ilankovan A, Morrison B III 2010 Age-dependent regional mechanical properties of the rat hippocampus and cortex. J. Biomech. Eng. 132, 011010 (doi:10.1115/1.4000164) 20524748 10.1115/1.4000164

[26] House DR, Elstrott J, Koh E, Chung J, Feldman DE 2011 Parallel regulation of feed forward inhibition and excitation during whisker map plasticity. Neuron 72, 819–831. (doi:10.1016/j.neuron.2011.09.008) 22153377 10.1016/j.neuron.2011.09.008 PMC3240806

[27] Hutter JL, Bechhoefer J 1993 Calibration of atomic-force microscope tips. Rev. Sci. Instrum. 64, 1868–1873. (doi:10.1063/1.1143970)

[28] Soza G, Grosso R, Nimsky C, Hastreiter P, Fahlbusch R, Greiner G 2005 Determination of the elasticity parameters of brain tissue with combined simulation and registration. Int. J. Med. Robot. Comput. Assist. Surg. 1, 87–95. (doi:10.1002/rcs.32) 10.1002/rcs.32 17518395

[29] Gefen A, Gefen N, Zhu Q, Raghupathi R, Margulies SS 2003 Age-dependent changes in material properties of the brain and braincase of the rat. J. Neurotrauma 20, 1163–1177. (doi:10.1089/089771503770802853) 14651804 10.1089/089771503770802853

